# Infliximab Therapy Leading to Pulmonary Tuberculosis in a Patient With Negative Interferon γ Release Assay (IGRA)-Based QuantiFERON Gold Test

**DOI:** 10.1177/2324709617717540

**Published:** 2017-07-06

**Authors:** Soni Parita, Kumar Vivek, Kamholz Stephan, Pascal William, Kupfer Yizhak

**Affiliations:** 1 Maimonides Medical Center, Brooklyn, NY, USA

**Keywords:** tuberculosis, Crohn’s disease, lung cancer (oncology), infliximab therapy, QuantiFERON gold test, medical education

## Abstract

Infliximab therapy is associated with higher rates of active tuberculosis (TB), particularly extrapulmonary and disseminated forms with unusual symptoms. We report the case of a 66-year-old man with Crohn’s disease who developed TB mimicking lung cancer on imaging. He presented with cough and fever of 2 weeks’ duration shortly after starting infliximab. Computed tomography of the chest revealed a 7.0 × 3.2 cm^2^ pleural-based mass, highly suspicious for malignancy. Histopathological examination confirmed the diagnosis of TB. The mass disappeared after antitubercular treatment, and the patient recovered completely. A review of the literature suggests that TB masquerades as lung cancer clinically and radiologically. The classical lesions of TB are cavitatory with calcifications. Mass lesions without cavity or calcifications are rare and are mostly reported from regions endemic for TB. The majority of patients on infliximab therapy required biopsy for accurate diagnosis of TB because of its unusual presentation.

## Introduction

Infliximab is a monoclonal antibody against tumor necrosis factor α (TNF-α). It is FDA approved for many autoimmune conditions, including rheumatoid arthritis and Crohn’s disease.^1,2^ One of the many known side effects of infliximab therapy is reactivation of latent tuberculosis (TB).^3^ Because of the resemblances in clinical and radiological features, tubercular lesions in the lung may mimic malignancy. TB accounts for 27% of all infections initially presumed to be lung cancer on imaging studies.^4^ The characteristic radiological findings of either disease are not specific enough to differentiate between TB and lung cancer and frequently requires histopathological and microbiological testing for diagnosis.^5^ However, mass-like appearance on imaging is rare, and most of these cases are reported from TB endemic regions.^6^ In the United States, TB is mostly seen in the immigrant population or in patients who are immunocompromised because of human immunodeficiency virus (HIV) or immunosuppressive therapy.

We present a case of an elderly patient with Crohn’s disease on infliximab therapy, who presented with a pleural-based mass, initially mimicking lung cancer. His sputum for acid fast bacilli culture and smear was negative. He was negative for latent TB on interferon γ release assay (IGRA)-based QuantiFERON gold test. The diagnosis of TB was confirmed on the biopsy of the lung mass. The mass disappeared as soon as the patient was started with anti-TB treatment.

## Case Presentation

A 68-year-old African American man with history of prostate cancer, hypertension, and bipolar disorder was brought to the emergency room with lethargy, cough with brownish sputum, and fever for 3 days. He also complained of left-sided chest pain that was pleuritic in nature.

In 2015, he was admitted with complaints of abdominal pain, bloody diarrhea, and weight loss and was diagnosed to have Crohn’s disease. He was treated with mesalamine. Since then he had had multiple admissions with intractable abdominal pain and diarrhea and underwent colectomy with formation of colostomy in September 2015 for pancolitis. After surgery, his abdominal pain improved, but he continued to have bloody diarrhea. His postoperative hospital course was complicated by the development of rectal abscess and fistula. Because of his intractable disease, he was started on infliximab therapy.

The patient was born in the United States without any history of traveling outside the country or TB in the past. He denied recent exposure to any patient with active TB. He also denied consumption of any type of unpasteurized dairy products, such as milk or cheese. He was screened for latent TB with a QuantiFERON gold test before initiation of infliximab therapy. The result from the QuantiFERON gold test revealed mitogen 2.23 IU/mL and adjusted TB antigen 0.03 IU/mL, and nil was 0.08 IU/mL. Based on this result, the QuantiFERON gold test was deemed negative. He received infliximab at a dose of 5 mg/kg body weight, scheduled at the zeroth, second, and eighth weeks followed by every eighth week for 6 months. He received 2 doses of infliximab before this admission.

During this admission, he complained of cough with brownish sputum for 3 days. His diarrhea and abdominal pain were significantly better now, but he reported significant weight loss (~30 lbs) in the past 2 months. He was a nonsmoker. His medications included mesalamine, infliximab, and lamotrigine.

On physical examination, he was a malnourished, ill-appearing, and cachectic man weighing 61 kg, with a body mass index of 21.8 kg/m^2^. He was febrile, with a temperature of 103.1°F, heart rate 121/min, respiratory rate 26/min, blood pressure 121/84 mm Hg, and SpO_2_ 95% with 3 L of oxygen via nasal cannula. Lung examination revealed crepitation over the left infraclavicular region. Cardiac examination was normal. Abdominal examination was significant for the presence of a scar from the previous surgery and colostomy bag on the left lower quadrant with brown nonbloody stool. Hemoccult stool test was negative.

## Investigations

The initial laboratory investigations revealed leucocytosis 12.1 × 10^3^/UL, haemoglobin/haematocrit (Hgb/Hct) 12.9/38.8, with normal renal and liver functions. Anti-*Saccharomyces cerevisiae* antibodies IgG and IgA were positive. Urinalysis was negative, and cardiac enzymes were normal. The patient’s sputum was obtained and sent for acid fast bacilli culture and smear. The sputum was collected 4 times on 4 different days. The test revealed negative result on all the 4 occasions. Blood and urine cultures were also negative. Echocardiogram revealed no vegetation.

**Figure 1.**
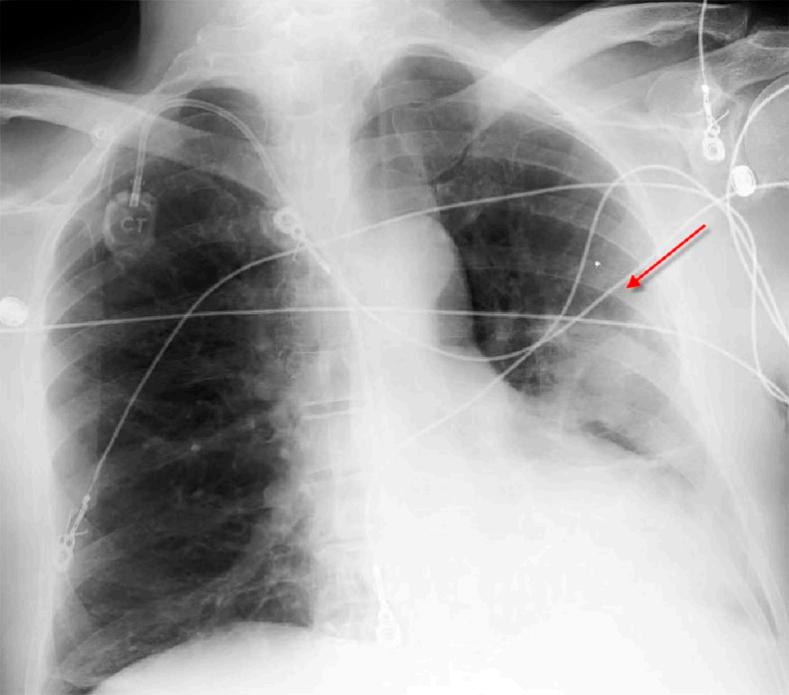
Posteroanterior view of the chest radiograph showing minimal pulmonary vascular congestion with left upper lobe opacity.

**Figure 2.**
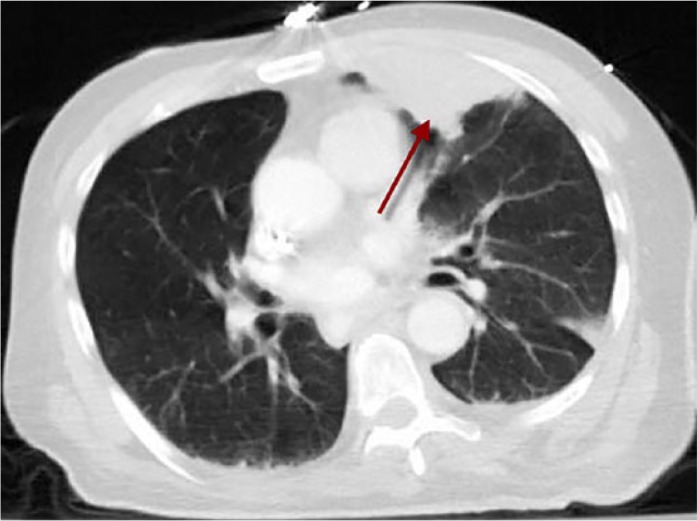
Computed tomography scan of the chest showing a 7.0 × 3.2 cm^2^ pleural-based left upper lobe soft-tissue mass.

A posteroanterior view of the chest radiograph showed minimal pulmonary vascular congestion with a left upper lobe opacity (Figure 1). No hilar adenopathy or pleural effusions were noted. The computed tomography (CT) scan of the chest showed a 7.0 × 3.2 cm^2^ pleural-based left upper lobe soft-tissue mass suspicious for neoplasm (Figure 2). There were no signs of lymphadenopathy, cavitation, calcification, or consolidation on the CT scan of the chest.

He was initially admitted with the diagnosis of sepsis secondary to health care–associated pneumonia and workup of possible lung tumor. Because of his poor functional status, malnutrition, and poor candidacy for surgical or chemotherapeutic intervention, the implications of biopsy with its risks and benefits were extensively discussed with the patient. However, he opted for complete workup at this point. He then underwent CT-guided biopsy of the left upper lobe lung mass.

**Figure 3.**
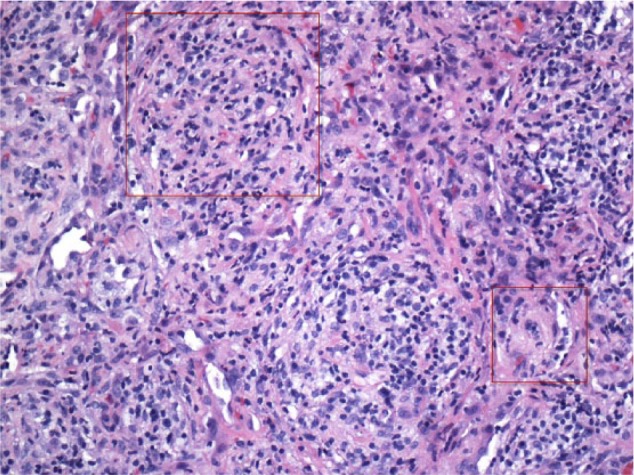
Biopsy of the lung mass showing noncaseating granuloma with nodularities composed of histiocytes.

**Figure 4.**
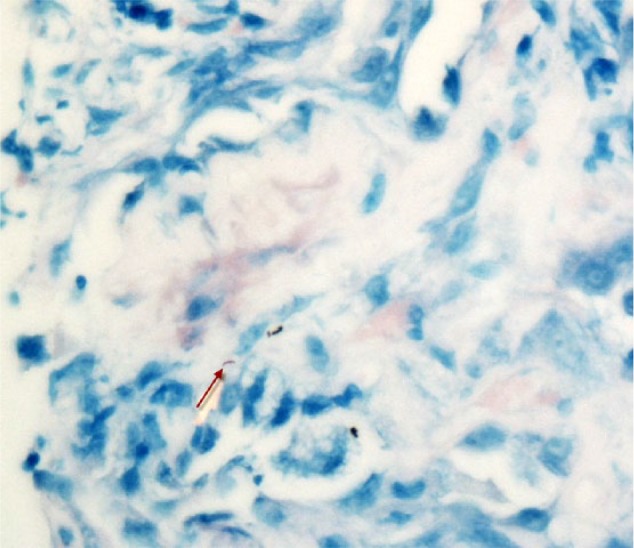
Biopsy of the lung mass showing nonnecrotizing granulomatous tissues positive for acid-fast organisms (Ziehl-Neelsen).

The histopathological examination of the biopsied sample revealed nonnecrotizing granulomatous tissues positive for acid-fast organisms (Ziehl-Neelsen; Figures 3 and 4). No evidence of vasculitis or tumor was seen. Special stains (Grocott methenamine silver) for fungi, including *Pneumocystis carinii*, were also negative.

## Differential Diagnosis

The most likely differential diagnosis of the mass-like lesion in this patient was malignancy, most likely primary lung cancer. Other less likely causes included infectious processes such as fungal infections and TB. A specific diagnosis could not be made based on radiographic findings. The presence of cough and weight loss with findings on CT scan of the chest (pleural-based mass without cavity and calcification) suggested the diagnosis of lung cancer as the most likely differential diagnosis.

However, TB can also present with similar clinical features. Pulmonary TB can affect both the upper and lower lobes; the upper lobe is typically involved during reactivation of latent TB, whereas primary TB lesions are associated with calcifications, mostly in the lower lobes.^7^ Infliximab treatment is associated with the increased risk of extrapulmonary TB.^3,7^ In this case, the negative history of TB in the past without any recent exposure and negative QuantiFERON gold test less than 8 weeks prior made the diagnosis of TB less likely. Moreover, lesions typical of TB, such as cavity or calcifications, were absent on the CT scan of the chest.

## Treatment

After establishment of diagnosis of TB, the patient was immediately shifted to an air-borne isolation unit; infliximab therapy was stopped; and he was treated with rifampin, isoniazid, pyrazinamide, and ethambutol (RIPE therapy).

## Outcome

**Figure 5.**
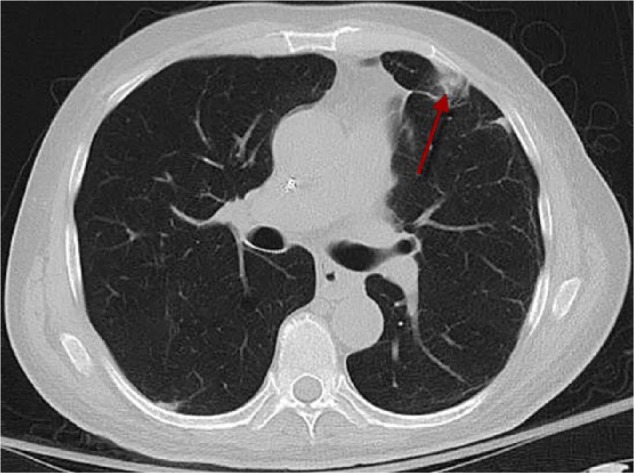
Repeat computed tomography scan of the chest after rifampin, isoniazid, pyrazinamide, and ethambutol therapy showing significant improvement in the size of the mass.

After the first 7 days of RIPE therapy, a repeat CT scan of the chest was performed, which revealed significant improvement in the size of the mass, significant for good response to antitubercular therapy (Figure 5).

The department of health was informed. Serial CT scans were conducted because of the high suspicion of malignancy based on its appearance on initial imaging. However, serial CT revealed complete resolution of the mass lesion on subsequent imaging. After 14 days of RIPE therapy and close monitoring, he was discharged back to the nursing home in a stable condition. It was recommended that he receive RIPE therapy for 6 months.

## Discussion

The unusual presentation of TB is especially concerning in countries with lower rates where risk of misdiagnosis is higher because of low suspicion. The resurgence of TB in developed countries is attributed to HIV infection, transplant immunosuppression, and treatment with steroids or TNF-α blockers. TNF-α blockers such as infliximab are associated with reactivation of TB based on data from postmarketing surveillance reports.^3^ According to this report, 70 patients developed TB after a median time period of 12 weeks after starting infliximab, with the majority developing extrapulmonary TB.^3^ Of note, the screening for latent TB prior to initiation of infliximab therapy was not mandatory at that time. It is not known if the risk still persists after more meticulous screening for latent TB, with newer interferon γ released assays before starting treatment with infliximab.

The classical presentation of pulmonary TB on chest imaging includes consolidation with irregular margins, pleural effusion, and thick wall cavities, which can also be seen in lung cancer.^8^ Pitlik et al^9^ reported 26 cases among more than 70 000 patients from the Texas cancer center during a 10-year period (1973-1982), who were referred with presumptive diagnosis of neoplasm but actually had bacteriologically proven TB without any evidence of malignancy. Classic symptoms of TB, such as fever, hemoptysis, and weight loss, were uncommon.^9^ Multilobar infiltration, upper lobe infiltrates, and pleural effusion were the most common abnormalities on chest radiograph.^9^ Laboratory abnormalities were unusual.^9^

Conversely, mass-like appearance without any calcification or cavitatory lesions is rarely described from countries with a low incidence of TB. The mass-like presentations of TB are mostly reported from TB-endemic regions. The common reasons for mass-like appearance in decreasing order are endobronchial TB, tuberculous pneumonia, conglomerated lymph nodes, or parenchymal granulomatous formations.^10,11^ Rana et al,^6^ in a case series, described 6 TB patients with mass-like lesions from a TB-endemic region. However, unlike the present case, mass-like tubercular lesions were localized more in the right lower lobe. Thus, the diagnosis remains unclear after imaging and should be confirmed by pathological and microbiological tests.

Moreover, our patient was negative for latent TB on IGRA-based QuantiFERON gold test, which was done 2 months prior to starting infliximab. He developed TB after 4 weeks of infliximab treatment, with left upper lobe involvement. In the absence of recent exposure to a patient with active TB, his disease most likely resulted from reactivation rather than a new infection. The performance of IGRA-based assays in diagnosing latent TB in patients with Crohn’s disease is not clear. Infliximab is used in patients with more debilitating disease with lack of response to frontline therapies.^12^ Such patients are frequently malnourished and show anergy to TB screening test, leading to false negative tests.

In conclusion, differentiating pulmonary TB from neoplasms can be challenging clinically and radiologically. Rarely, TB may present as a mass-like lesion on imaging, mimicking malignancy.^13^ The diagnosis is more difficult in patients on immunosuppressive therapy because of atypical presentation of TB. With poor nutrition resulting from debilitating disease, these patients may be falsely negative on initial screening for latent TB at the outset of infliximab therapy. Active surveillance for symptoms of active TB with lower threshold for workup may aid in timely diagnosis and prevent catastrophic outcomes. Despite the higher diagnostic accuracy of newer IGRAs in diagnosing latent TB, infliximab therapy is associated with reactivation of TB, and every effort should be taken to rule out TB during the further workup.

## Conclusion

The differentiation of TB from lung cancer based on radiological features alone may be challenging and frequently ambiguous,^14^ thereby requiring pathological diagnosis in a majority of patients. Newer IGRAs may be falsely negative particularly in patients with Crohn’s disease because they are frequently debilitated and malnourished. Active surveillance for symptoms of active TB while on infliximab therapy is mandatory for early diagnosis to prevent catastrophic outcomes. The presentation of TB in patients on infliximab therapy is frequently atypical and often requires tissue examination for accurate diagnosis. Because these patients are at higher risk for active TB, every measure should be taken to rule out TB in these patients.
